# The Role of MHC-II Diversity over Enclosure Design in Gut Microbiota Structuring of Captive Bengal Slow Lorises

**DOI:** 10.3390/biology14081094

**Published:** 2025-08-21

**Authors:** Rong Jiang, Xiaojia Zhang, Lei Xie, Yan Zhang, Changjun Zeng, Yongfang Yao, Huailiang Xu, Caoyang Yang, Xiao Wang, Qingyong Ni, Meng Xie, Chuanren Li

**Affiliations:** 1 Key Laboratory of Livestock and Poultry Multi-Omics, Ministry of Agriculture and Rural Affairs, College of Animal Science and Technology, Sichuan Agricultural University, Chengdu 610000, China; rong106412@163.com (R.J.); xl2449616395@outlook.com (L.X.); yanzhang@sicau.edu.cn (Y.Z.); zengchj@sicau.edu.cn (C.Z.); niqy@sicau.edu.cn (Q.N.); 2 Farm Animal Germplasm Resources and Biotech Breeding Key Laboratory of Sichuan Province, College of Animal Science and Technology, Sichuan Agricultural University, Chengdu 610000, China; 3 College of Life Science, Sichuan Agricultural University, Yaan 625000, China; zzz0995@outlook.com (X.Z.); 13603@sicau.edu.cn (Y.Y.); xuhuail@sicau.edu.cn (H.X.); 4 Sichuan Ganzi Ecological Environment Monitoring Center, Ganzi 626700, China; ycy37@outlook.com; 5 Sichuan Minzu College, Ganzi 626700, China; wx744@outlook.com

**Keywords:** *DRB1e2*, polymorphisms, genotypes, conservation, nocturnal primates

## Abstract

The endangered Bengal slow loris (*Nycticebus bengalensis*) relies heavily on captive/rescue populations for conservation. This study investigated the critical link between Major Histocompatibility Complex (MHC) class II DRB1 exon 2 (*DRB1e2*) genetic variation and gut microbiota in 46 captive individuals, aiming to improve ex situ management. Using standardized conditions across three enclosure types, we characterized *DRB1e2* polymorphism via targeted sequencing and analyzed fecal microbiota using 16S rRNA gene amplicon sequencing. Results demonstrated that high *DRB1e2* polymorphism significantly reduced microbial community evenness. Specific genotypes showed distinct microbial associations: G9 strongly correlated with beneficial short-chain fatty acid producers like *Fructobacillus*, and G2 positively correlated with *Bifidobacterium* spp., while G2, G3, and G4 correlated negatively with *Buchnera* (a nutrient-provisioning symbiont). Genotypes and polymorphism collectively explained 9.77% of microbiota variation, exceeding the weaker (5.15%), though significant, influence of enclosure type on β-diversity. These findings reveal that host *DRB1e2* variation is a primary driver shaping gut microbiota structure and taxon abundance in captive slow lorises, providing evidence for MHC-mediated host–microbe co-adaptation. This offers a genetically informed framework for optimizing conservation strategies, such as tailoring diets or probiotics to specific genotypes, to enhance gut health and population viability.

## Simple Summary

The Bengal slow loris is an endangered primate species, so the management of its captivity for rescue purposes is of great significance. Here, we explored the connection between MHC genetics and the intestinal microbiota of captive Bengal slow lorises. Based on genetic research findings, we discovered that although environmental regulation plays a significant role, the host genetic factors still have a dominant influence. This integrated genetic–microbial framework significantly advances conservation strategies for this endangered primate. When providing protection strategies for the captive population, combining genotype-targeted intervention measures with optimized captive breeding designs helps to enhance microbial diversity while balancing the risk of pathogens.

## 1. Introduction

The intricate symbiotic relationship between hosts and their gut microbiota represents a cornerstone of organismal biology, with profound implications for host metabolism, immune function, and overall fitness. Mounting evidence underscores that gut microbial communities are dynamically shaped by the interplay of environmental factors (e.g., diet, habitat) and host genetics [1,2,3]. Among genetic determinants, the Major Histocompatibility Complex (MHC) class II genes, particularly the hypervariable exon 2 region of the DRB locus (*DRB1e2*), play a pivotal role in adaptive immunity by presenting pathogen-derived peptides to CD4+ T cells [4,5]. This antigen presentation capability directly influences host immune responses and indirectly sculpts gut microbiota composition through selective pressure on microbial colonization and persistence—a phenomenon termed the “MHC-mediated microbiome sculpting hypothesis” [6,7].

Recent advances have illuminated the bidirectional co-evolution between MHC polymorphism and gut microbiota. High MHC diversity enhances the host’s capacity to recognize diverse microbial antigens, thereby promoting microbiota richness and stability while suppressing pathobiont expansion [8,9]. For instance, MHC-II heterozygosity correlates with increased abundance of beneficial *Bifidobacterium* and reduced pathogen loads in lemurs [10,11]. Conversely, low MHC diversity—common in small, fragmented populations—can disrupt microbial homeostasis, leading to dysbiosis and heightened disease susceptibility [12]. This genetic–microbial crosstalk is now recognized as a critical adaptive mechanism in wildlife facing environmental stressors [13,14].

Despite this conceptual progress, critical gaps persist in non-model and endangered primates. The Bengal slow loris (*Nycticebus bengalensis*), an IUCN Endangered nocturnal primate endemic to Southeast Asia, faces catastrophic declines due to habitat fragmentation and illegal wildlife trade [15,16]. Confiscated individuals increasingly rely on ex situ breeding programs, yet captivity imposes profound physiological stressors. Artificial diets, reduced microbial exposure, and chronic confinement alter gut microbiota, manifesting as reduced diversity, loss of keystone taxa (e.g., fiber-degrading Firmicutes), and proliferation of opportunistic pathogens [17,18]. Crucially, the role of MHC genetics in modulating these captivity-induced dysbioses remains entirely unexplored in slow lorises—a significant oversight given the MHC’s established role in microbial resilience.

## 2. Materials and Methods

### 2.1. Animal Ethics Statement

Sample collection and animal experiments were approved by the Institutional Review Board (IRB13627) and the Institutional Animal Care and Use Committee of the Sichuan Agricultural University, China, under permit number 20240098, as well as the Administration for Wild Animal Protection in Yunnan Provinces, China, and adhered to the American Society of Primatologists Principles for the Ethical Treatment of Non-Human Primates. This article has followed the Arrive guidelines.

### 2.2. Sample Collection and DNA Extraction

Fecal samples were collected from 46 captive Bengal slow lorises at Dehong Wildlife Rescue Center, China (24.38287° N, 98.45872° E), during July 2022. To ensure sample integrity, clean trays were placed under cages prior to morning feeding, preventing cross-contamination and environmental exposure. All subjects originated from confiscated/rescued wild populations with 12–18 months of captivity. Individuals were stratified into three groups by enclosure type: Group I (*n* = 23), single occupancy in standard cages (0.58 × 0.43 × 0.46 m^3^); Group II (*n* = 15), single occupancy in compact cages (0.48 × 0.30 × 0.33 m^3^); Group III (*n* = 8), enriched enclosures (4 m^2^ floor area, 2.5 m height; climbing structures; 1–2 individuals/room). The following ambient parameters were standardized across groups: temperature (25 ± 1 °C), humidity (60 ± 5%), and diet (rice/fruits/locusts). Variations were strictly limited to enclosure dimensions and social housing. Fresh fecal samples were immediately transferred to labeled centrifuge tubes, flash-frozen on dry ice, and stored at −80 °C at Sichuan Agricultural University.

Genomic DNA was extracted from fecal samples using the TiaNamp Stool DNA Kit (Tiangen Biotech Co., Ltd., Beijing, China) according to the manufacturer’s instructions. The extracted DNA was assessed for quality using 0.8% agarose gel electrophoresis and quantified using NanoDrop 2000 (Thermo Scientific, Wilmington, DE, USA). Subsequently, the DNA was stored at −20 °C for further experiments.

### 2.3. DRB1 Exon 2 Gene Sequencing and Allele Calling

Targeted primers for *DRB1e2* exon 2 (Forward: 5′-GTTGTGTCTGCACACCGT-3′; Reverse: 5′-GCAGGCTAAGTTTGAGTGT-3′) were designed using Primer-BLAST (NCBI). Primer specificity was validated against the Bengal slow loris reference genome (NCBI Bioproject: PRJNA785018) and homologous sequences from related primates [19,20]. PCR reactions (50 µL volume) contained 25 µL 2× SanTaq Master Mix, 2 µL of each primer, and 10–50 ng DNA template. Amplification conditions were as follows: initial denaturation at 94 °C (5 min); 30 cycles at 94 °C (10 s), 52.5 °C (20 s), 72 °C (30 s); and final extension at 72 °C (5 min). Eight technical replicates per sample ensured ≥95% allele detection accuracy.

PCR products were purified (SanPreo Column DNA Kit), cloned into pUCm-T vectors, and subjected to bidirectional Sanger sequencing using Applied Biosystems™ 3730XL (Thermo Fisher Scientific Inc., Waltham, MA, USA). Forward and reverse reads were trimmed off primers/adapters using Fastp v0.20.0 and quality-filtered (Phred score > 20, Q > 80). The quality-controlled sequences were assembled into contiguous sequences via Seqman v7.1.0 and translated to amino acid sequences in MEGA v11 to confirm reading frame integrity.

Despite being optimized for shotgun data, the TARGT pipeline [21] was adapted for Sanger-derived FASTA files due to cross-platform compatibility and enhanced accuracy for low-variation targets. The pipeline output was validated against Kennedy’s criteria: alleles required either (a) presence in ≥2 individuals or (b) ≥3 identical clones in one individual [22].

### 2.4. Polymorphism and Phylogenetic Analysis

Population genetic analyses were conducted on *DRB1e2* sequences stratified by the three captive groups (I–III). Using DNAsp v6.12.03 [23], polymorphic information sites and nucleotide diversity (π) were calculated, pairwise Fst between groups was quantified to assess genetic differentiation, and Tajima’s D for each group was computed to evaluate neutrality deviations. Variant calling was performed with Samtools v1.19.2 to categorize nucleotide substitution types. Genotype data were analyzed in R v4.3.2 (adegenet package) to determine allele frequencies, individual heterozygosity (H_E_), effective allele number (A_E_), and polymorphic information content (PIC). Allele frequency distributions were visualized using ggplot2. Hardy–Weinberg equilibrium was tested (SNPassoc package), and Kruskal–Wallis ANOVA compared median H_E_ and π values across groups.

A phylogenetic analysis was performed to assess the specificity of *DRB1e2* amplification products. Using known alleles from closely related species (Table 1) as outgroups, we constructed a maximum-likelihood (ML) tree in MEGA v11 to classify obtained *DRB1e2* alleles and validate primer specificity for the target locus (i.e., confirm amplification of *DRB1* paralogs versus other DRB genes). Given the limitations of single-copy alleles and highly variable exonic regions, this phylogeny serves primarily to evaluate PCR target specificity, not to infer interspecies evolutionary relationships. The best-fit nucleotide substitution model (T92 + G) was selected under the Akaike Information Criterion (AIC). Branch support was assessed with 1000 bootstrap replicates; only nodes ≥ 50% are shown.

### 2.5. Microbial Diversity and Correlation Analysis

Total genomic DNA was extracted from fresh fecal samples, followed by amplification of the bacterial 16S rRNA V3-V4 hypervariable region using universal primers 338F (5′-ACTCCTACGGGAGGCAGCA-3′) and 806R (5′-GGACTACHVGGGTWTCTAAT-3′). Purified PCR products were normalized, constructed into Illumina sequencing libraries, and subjected to paired-end sequencing (2 × 250 bp) on the NovaSeq 6000 platform (Illumina Inc., San Diego, CA, USA) after rigorous quality control. Raw sequencing reads underwent initial quality filtering with Trimmomatic (v0.33) to remove low-quality bases (Phred score < 20), followed by adapter trimming using Cutadapt (v1.9.1). Processed reads were merged and filtered for chimeric sequences via USEARCH (v10.0), generating high-fidelity sequences for downstream analysis. Operational taxonomic units (OTUs) were clustered at 97% similarity threshold using USEARCH and filtered at 0.005% relative abundance to eliminate spurious taxa. Taxonomic classification was performed against the SILVA v138 reference database through QIIME2’s (v2020.6) classify-sklearn algorithm, yielding abundance tables across six taxonomic ranks (phylum to species). Microbial α-diversity was quantified using Chao1, abundance-based coverage estimator (ACE), Shannon, Simpson, and Faith’s phylogenetic diversity (PD) indices in QIIME2, with group-wise comparisons assessed via Kruskal–Wallis tests. For β-diversity analysis, Jaccard, Bray–Curtis, and (un)weighted UniFrac distance matrices were computed and visualized through principal coordinate analysis (PCoA), non-metric multidimensional scaling (NMDS), and redundancy analysis (RDA), while permutational multivariate ANOVA (PERMANOVA; 999 iterations) decomposed variance contributions. Differential microbial abundance was identified using LEfSe (https://usegalaxy.org/) on the Galaxy platform (LDA score > 2.0, *p* < 0.05), with the top 20 discriminant genera further analyzed in Spearman correlation heatmaps to elucidate associations between *DRB1e2* genotypes, enclosure types, and beneficial bacterial taxa.

## 3. Results

### 3.1. DRB1e2 Genetic Diversity

**Figure 1:**
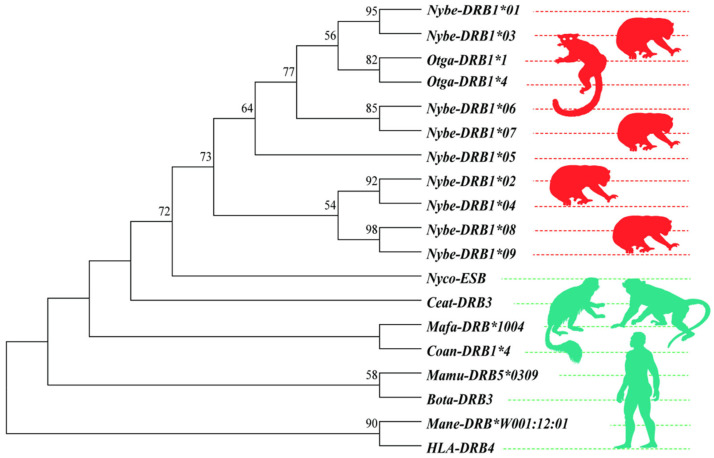
Phylogenetic tree of *DRB1e2* alleles in Bengal slow lorises.

Nine distinct *DRB1e2* alleles (designated Nybe-DRB1*01–09) were identified in the captive Bengal slow loris population (Supplementary Table S1). The Nybe-DRB1*06 allele exhibited a unique three-nucleotide insertion, adding a proline residue at position 9 of the translated protein (Supplementary Table S2). Phylogenetic analysis confirmed all amplified sequences belonged exclusively to the *DRB1* clade, showing clear divergence from paralogous *DRB3*, *DRB4*, and *DRB5* genes (Figure 1). In silico PCR validation (Primer-BLAST) confirmed target specificity with no off-target amplification.

Individuals carried 1–6 alleles (mean ± SD: 2.62 ± 1.42), with differential allele frequencies: Nybe-DRB1*05 demonstrated the highest prevalence, while Nybe-DRB1*08 occurred only in individual N53. Eleven genotypes (G1–G11) were identified, with G3 (Nybe-DRB1*02/04/05) being predominant (20 individuals, f = 0.43), followed by the homozygous G2 (Nybe-DRB1*03) (14 individuals, f = 0.30). Seven singleton genotypes were excluded from comparative analyses (Supplementary Table S3).

Population-level metrics revealed low heterozygosity (H_E_ = 0.29) but high polymorphism (PIC = 0.82; allelic richness A_E_ = 5.71). Nucleotide diversity (π) varied across the exon, peaking at 0.19 within the 27–58 bp segment. The negative Tajima’s D (−0.21, *p* < 0.05) and near-zero Fst (−0.01) indicated minimal population subdivision with greater variation within than between groups.

### 3.2. Group-Specific Variation

**Figure 2:**
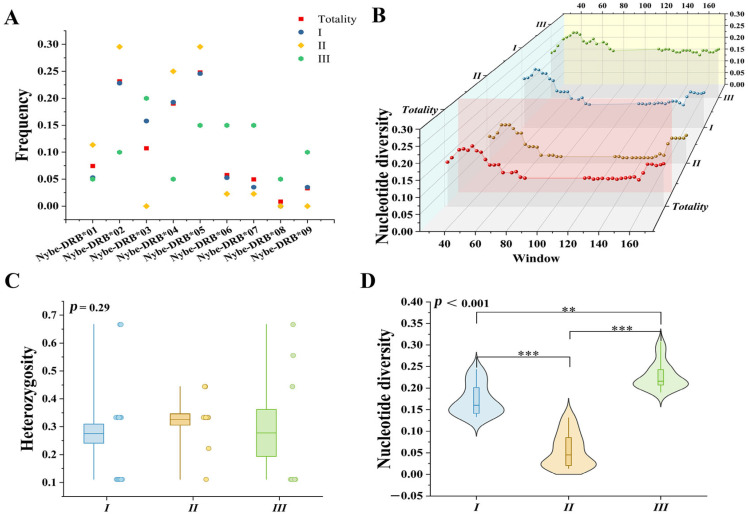
Distribution of *DRB1e2* gene frequencies and polymorphism comparison in captive populations and different enclosures. (**A**) Distribution of *DRB1e2* gene frequencies, red squares represent “Totality,” while blue circles, yellow diamonds, and green triangles denote groups I, II, and III, respectively; (**B**) nucleotide polymorphism curves of *DRB1e2,* red, blue, brown, and green dots with shaded areas correspond to “Totality,” group I, group II, and group III in terms of nucleotide diversity; (**C**) comparison of heterozygosity, light blue, tan, and light green boxplots with scatterplots represent groups I, II, and III; (**D**) comparison of nucleotide polymorphism, light blue, tan, and light green violin plots are used to depict groups I, II, and III (**: *p* < 0.01; ***: *p* < 0.001).

Allele/genotype distributions differed significantly among enclosure groups (Figure 2; Supplementary Table S1). Nybe-DRB1*05 dominated Groups I and II, while Nybe-DRB1*03 reached the highest frequency in Group III. G3 was most frequent in Groups I and II (*n* = 10 each), and G2 predominated in Group III (*n* = 5). This distribution suggests potential social stress-mediated selection in co-housed Group III versus neutral drift in isolated Groups I/II. While heterozygosity (*p* = 0.24) and PIC (*p* = 0.24) showed no intergroup differences, nucleotide diversity varied significantly (*p* < 0.01): Group III > Group I > Group II (Table 2).

### 3.3. MHC–Microbiota Associations

**Figure 3:**
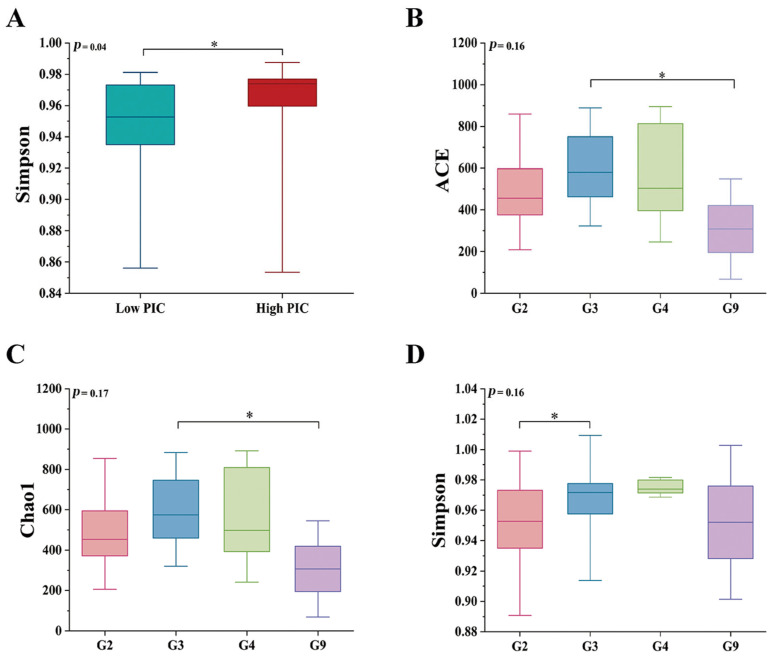
Comparison of α diversity indices under different polymorphisms (**A**) and genotypes (**B**–**D**) (*: *p* < 0.05). Abbreviations: PIC, polymorphic information; ACE, abundance-based coverage estimator.

*DRB1e2* polymorphism (PIC) showed no significant effect on microbiota richness (Chao1/ACE) or phylogenetic diversity (Supplementary Figure S1). However, microbial evenness—reflecting uniformity in species abundance distributions where low evenness indicates dominance by a few taxa and potential dysbiosis risk—exhibited a significant inverse relationship with polymorphism. Individuals with higher PIC displayed significantly lower evenness (Simpson index increase, *p* < 0.05; Figure 3A).

**Figure 4:**
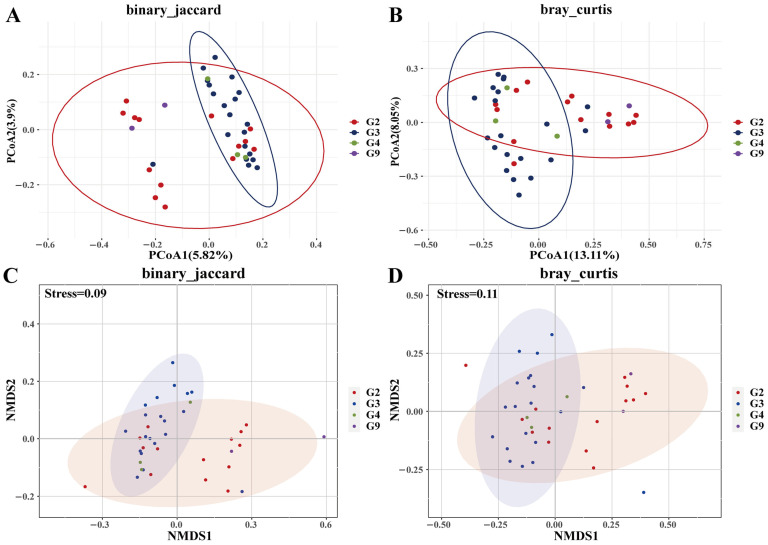
PCoA (**A**,**B**) and NMDS (**C**,**D**) analysis of β diversity (binary_jaccard and bray_curtis distances) under different genotypes of *DRB1e2.* In A, the red ellipse encompasses samples primarily classified as G2 (red dots), demonstrating clear clustering based on binary Jaccard dissimilarity. In B, the red ellipse also highlights samples mainly from group G2, though there is some overlap with other groups, reflecting partial intermixing under the Bray–Curtis dissimilarity measure. In C, samples within the ellipse belong to multiple groups (G3, G4, G9) in the NMDS ordination using binary Jaccard, indicating poorer separation compared to the PCoA result. In D, the ellipse again incorporates a mixture of groups (G2, G3, G4, G9) in the NMBS Bray–Curtis ordination, demonstrating broader overlap among groups. Collectively, the ellipses visually summarize the clustering patterns and dispersion of sample groups across multivariate ordination methods. Abbreviations: PCoA, principal coordinate analysis; NMDS, non-metric multidimensional scaling.

**Figure 5:**
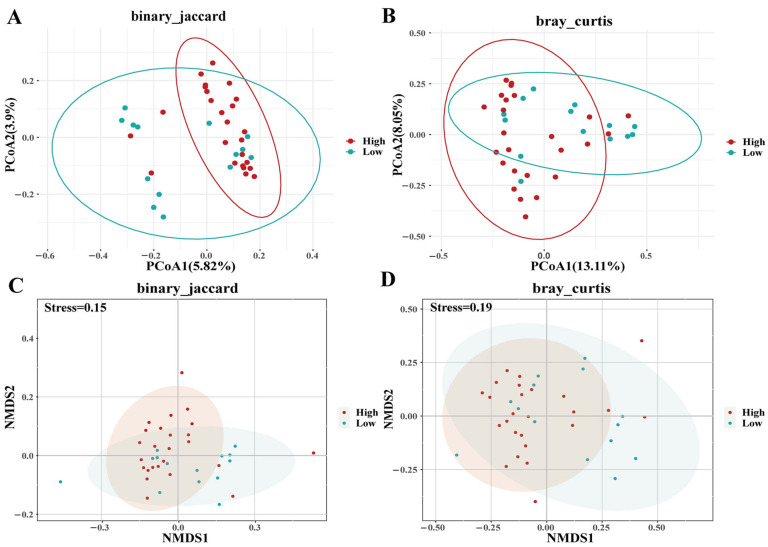
PCoA (**A**,**B**) and NMDS (**C**,**D**) analysis of β diversity (binary_jaccard and bray_curtis distances) under different polymorphisms of *DRB1e2.*

Genotype-specific microbial diversity patterns and relationships with PIC were observed. For example, G3 had higher ACE/Chao1 than G9 (*p* = 0.05), while G2 showed a lower Simpson index than G3 (*p* < 0.05) (Figure 3; Supplementary Figure S2). PCoA/NMDS with Jaccard (F_df_ = 1.13, *p* < 0.01, R_2_ = 0.09) and Bray–Curtis (F_df_ = 1.41, *p* < 0.01, R_2_ = 0.11) illustrated distinct clustering patterns for G3, with G2 proximity to G9, and G3 proximity to G4 (Figure 4), with no separation by (un)weighted UniFrac metrics (Supplementary Figure S3). High-PIC individuals clustered distinctly in Jaccard ordinations (F_df_ = 1.23, *p* < 0.01, R_2_ = 0.03) and Bray–Curtis (F_df_ = 1.65, *p* < 0.01, R_2_ = 0.04) (Figure 5). However, weighted and unweighted unifrac analysis did not differentiate between individuals (Supplementary Figure S4).

### 3.4. Genotype-Specific Microbiota Associations

**Figure 6:**
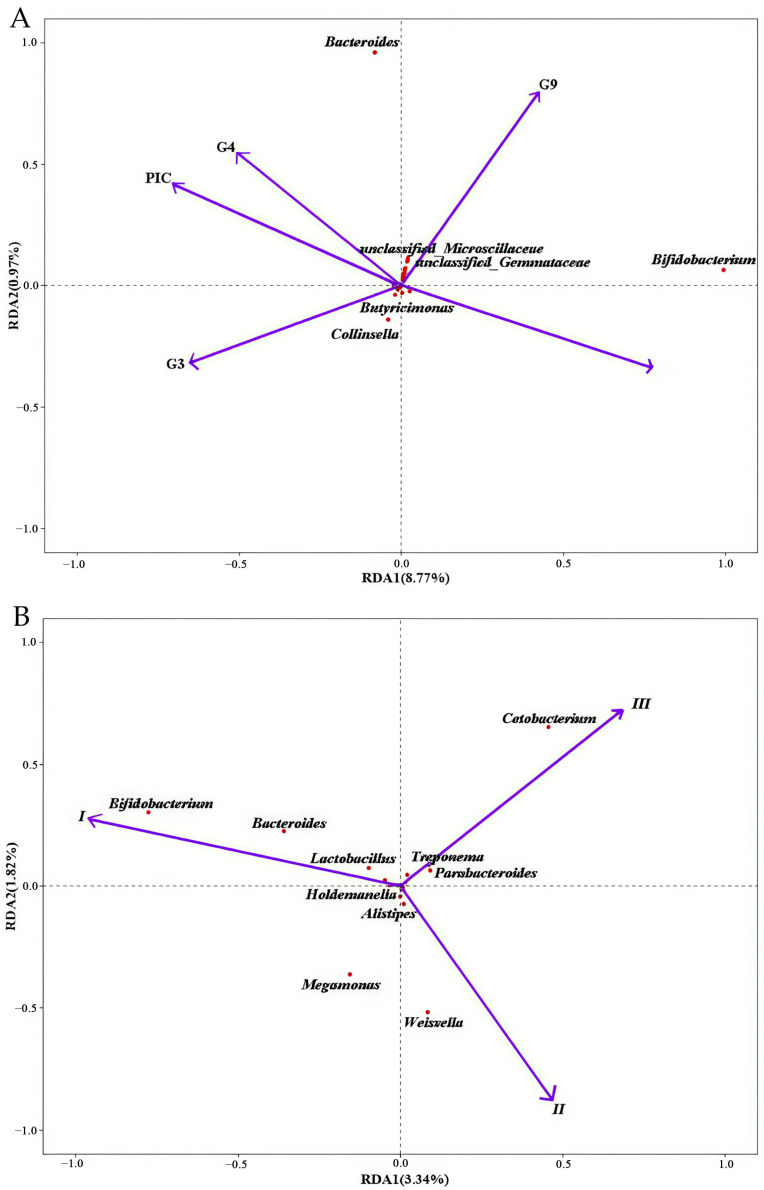
RDA plot showing the impact of genotypes and polymorphism (**A**) and captive environment on the intestinal microbiota composition of the Bengal slow loris (**B**). Bacterial genera are represented as red points. MHC variables and captive environment variables significantly influencing intestinal microbiota are indicated by arrows, marked in purple. For visualization purposes, only the 20 genera with the smallest *p*-values are labeled. Abbreviations: RDA, redundancy analysis; PIC, polymorphic information.

**Figure 7:**
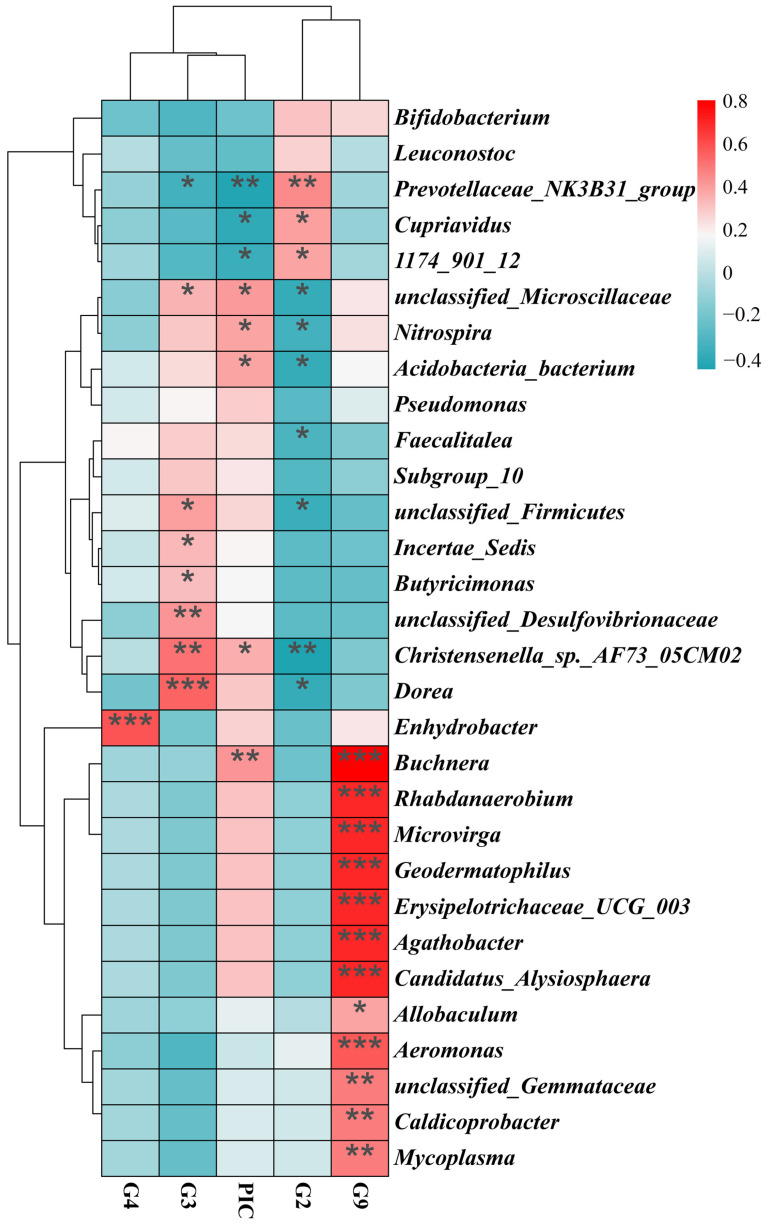
Correlation heatmap between *DRB1e2* genotypes and PIC with microbial genera. The heatmap depicts the estimated impact of the four *DRB1e2* genotypes on the 20 microbial genera with the smallest *p*-values within the group (Spearman). Blue indicates consistency between the presence of a given factor and lower relative abundance of a specific microbial genus, while red indicates that the genus is more common when this MHC factor is present. Black asterisks denote significant effects (* *p* < 0.05; ** *p* < 0.01; *** *p* < 0.001).

Multivariate analysis revealed that four *DRB1e2* genotypes (G2–G4, G9) combined with polymorphism (PIC) explained 9.77% of total microbiota variation, though the overall model lacked significance (*p* = 0.26; Figure 6). Redundancy analysis (RDA) ordination showed Axis 1 divergence driven by antagonistic effects of G2/G9 versus G3/G4/PIC and Axis 2 divergence separating G2/G3 from G4/G9/PIC. Regarding the microbiota composition, G4/G9/PIC was positively correlated with *Bacteroides*, while G2/G3 was negatively correlated with *Bacteroides*. Furthermore, G2/G9 had positive correlation with *Bifidobacterium* spp. (Figure 6). The genotype-specific signatures showed that *Buchnera* was significantly positively correlated with G9 but negatively correlated with G2/G3/G4, while an exclusively positive correlation was found between G4 and *Enhydrobacter*. Some antagonistic relationships were also observed. For example, G3 was negatively correlated with *Prevotellaceae*, but G2 showed positive correlation; G3 was positively associated with *Microscillaceae*, *Firmicutes*, *Dorea*, and *Christensenella*, all of which were negatively correlated with G2. G9 demonstrated broad positive associations with multiple bacterial genera, indicating a distinct microbiota-modulating profile (Figure 7).

### 3.5. Enclosure Effects on Microbiota

**Figure 8:**
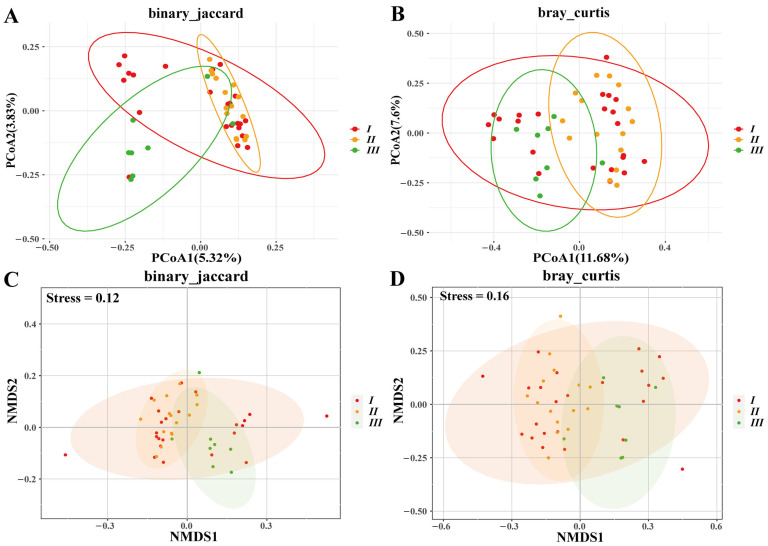
PCoA (**A**,**B**) and NMDS (**C**,**D**) analysis of β diversity (binary_jaccard and bray_curtis distances) under different enclosures. In A, the red ellipse delineates a distinct cluster of group I (red dots) under binary Jaccard dissimilarity. The orange and green ellipses enclose groups II and III, respectively, showing clear separation in the PCoA ordination. In B, the red ellipse again primarily contains group I, but with greater overlap with groups II and III under Bray–Curtis dissimilarity. The orange and green ellipses remain associated with groups II and III, though with reduced distinctness. In C, ellipses corresponding to groups I, II, and III illustrate moderate separation in the NMDS ordination with binary Jaccard. In D, all three group ellipses exhibit broader overlap in the NMDS Bray–Curtis ordination, reflecting weaker group discrimination.

**Figure 9:**
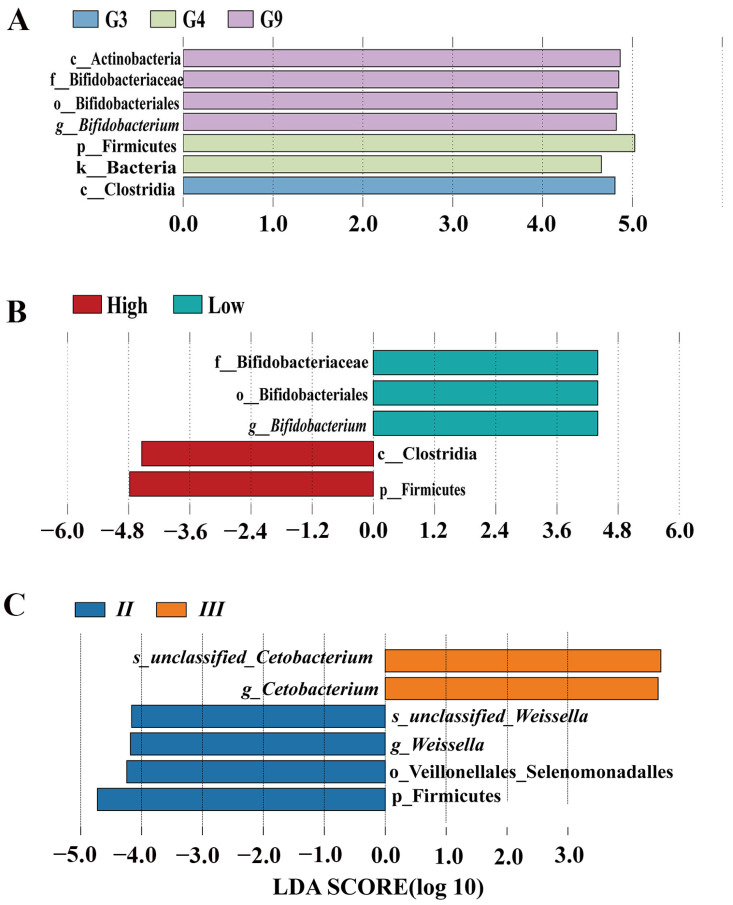
Linear discriminant analysis effect size (LefSe) analysis. The cladogram shows significantly different taxonomic groups of intestinal microbiota within groups of PIC (**A**), genotype (**B**), and captive environments (**C**) (LDA score > 4, *p* < 0.05). Species (s), genus (g), family (f), order (o), class (c), and phylum (p).

**Figure 10:**
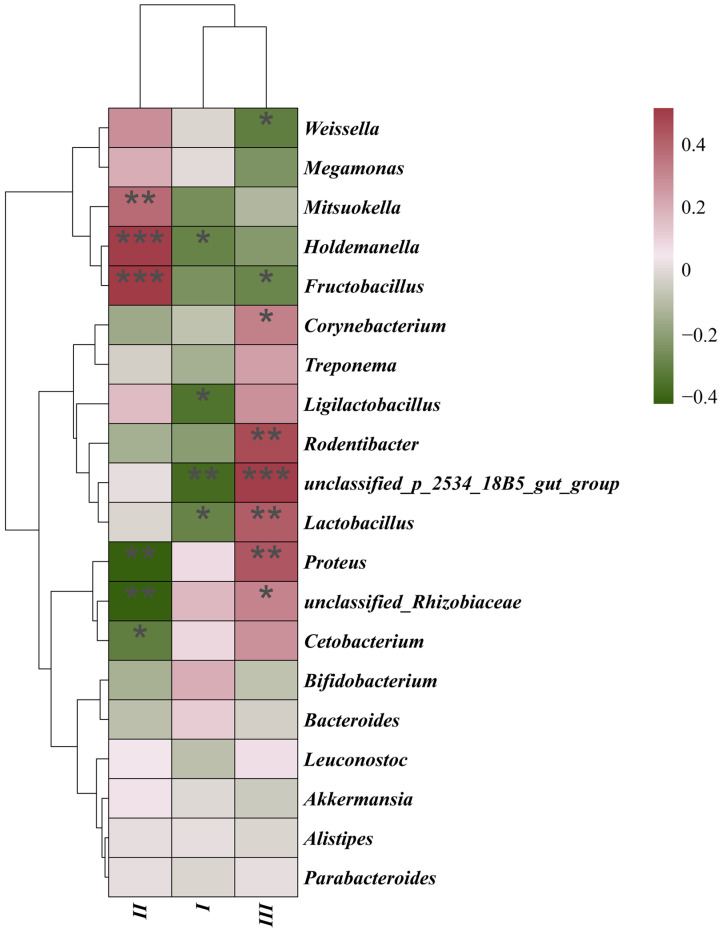
Correlation heatmap between different enclosures and microbial genera, showing the estimated impact size of three captive environments on the 20 microbial genera with the smallest *p*-values within groups (Spearman). Green indicates the presence of the given factor is consistent with lower relative abundance of specific microbial genera, while red indicates the genus is more common when this factor is present. Black asterisks denote significant effects (* *p* < 0.05; ** *p* < 0.01; *** *p* < 0.001).

While α-diversity metrics (species richness, evenness, phylogenetic diversity) showed no significant differences among enclosure groups (Supplementary Figure S5), β-diversity analyses revealed distinct microbial structuring. PCoA/NMDS ordinations (Jaccard/Bray–Curtis) demonstrated greater separation between Group II and III than between I/II or I/III (*p* < 0.01; Figure 8). Group III exhibited the most divergent community composition. In addition, genus-level differential abundance analysis identified key taxonomic shifts. For example, Group II was enriched in *Weissella*, and Group III was dominated by *Cetobacterium*. *Fructobacillus* showed the most pronounced abundance gradient across groups. Group II vs. III exhibited stronger differentiation than I vs. II or I vs. III (Figure 9). Group III showed significant positive correlations with multiple bacterial genera, while *Fructobacillus* and *Weissella* were notably reduced (Figure 10). Redundancy analysis attributed 5.15% of total microbiota variation to enclosure differences, though the global model remained non-significant (*p* = 0.27; Figure 6). Ordination patterns revealed distinct patterns: Axis 1, Group I opposed to II/III; Axis 2, Group II opposed to I/III.

## 4. Discussion

### 4.1. Association Between DRB1e2 and Microbial Community

The high *DRB1e2* polymorphism observed in captive Bengal slow lorises underscores its potential role in adaptive immune complexity. While our study cannot establish causality, the genotype-specific microbiota associations suggest coevolutionary dynamics, represented by allele-driven α-diversity modulation, bidirectional genotype–taxa relationships, and mechanistic knowledge gaps.

Elevated microbial richness in Nybe-DRB1*05 carriers (e.g., N1) implies allele-specific filtering of commensals, consistent with MHC-II-mediated microbial selection in primates [6]. Paradoxically, higher polymorphism reduced microbial evenness, potentially reflecting trade-offs between pathogen defense and symbiont maintenance. The antagonistic *Buchnera* responses (G2–G4 negative vs. G9 positive) mirror MHC-driven microbiota bifurcation [8]. As *Buchnera* supplies essential amino acids [24], G9-associated enrichment may enhance nutritional adaptation in captivity. G2’s strong correlation with *Bifidobacterium* spp.—a keystone genus in wild exudativorous primates [25]—highlights how host genetics interacts with dietary interventions [17] to shape beneficial taxa. The broad positive correlations of G9 with multiple genera may arise from its high allelic dosage (6 alleles), supporting the “gene dosage effect” hypothesis in MHC–microbiota crosstalk [26]. Though *DRB1e2* variants explained 9.77% microbiota variation, it inherently lacks the functional resolution to directly distinguish whether these shifts arise from this specific form of immune selection or from other potential mechanisms influenced by MHC, such as differential tolerance induction or indirect effects on gut physiology or inflammation. To unequivocally demonstrate the role of immune filtering via IgA targeting or similar adaptive immune mechanisms, future studies would greatly benefit from incorporating metatranscriptomic analysis.

### 4.2. Captive Environment as a Microbiota Modulator

The results unequivocally demonstrate that captive enclosure design restructures gut microbial communities in Bengal slow lorises, with β-diversity analysis revealing significant divergence in Group III (socially housed enriched enclosures) compared to Groups I/II (single-cage housing). This environmental effect manifests through two interconnected mechanisms: (1) social housing-mediated microbial exchange facilitated by direct conspecific contact, overriding genetic background limitations in microbial transmission [27] and (2) physical complexity-driven exposure diversity where climbing structures introduce novel environmental microbiota, altering colonization dynamics [18]. Taxon-specific shifts carry critical health implications: Group II exhibited *Weissella* enrichment—a fermentative symbiont enhancing nutrient utilization—while Group III showed dualistic alterations including beneficial *Cetobacterium* (vitamin B12 synthesis) and *Lactobacillus* augmentation (pathogen exclusion via bacteriocin production) [28] but concerning depletion of *Fructobacillus* (reducing SCFA output) and paradoxical enrichment of opportunistic pathogens (*Proteus* and *Corynebacterium* spp.) [29]. This “pathogen paradox” suggests environmental enrichment simultaneously increases pathogen exposure risk while enhancing microbial resilience through *Lactobacillus*-mediated competitive exclusion—a trade-off demanding careful management in conservation settings. Critically, when contextualized with our genetic findings, enclosures explained 5.15% of microbiota variance versus 9.77% from *DRB1e2* polymorphism, underscoring that while environmental modulation is significant [25], host genetics exerts predominant control. This necessitates integrated ex situ management strategies that pair genotype-targeted interventions (e.g., prioritizing high-heterozygosity individuals) with optimized enclosure designs balancing microbial diversity gains against pathogen risks.

## 5. Conclusions

This study establishes that MHC-II *DRB1e2* polymorphism (PIC = 0.82) is the primary driver of gut microbiota structure in captive Bengal slow lorises, explaining 9.77% of variation—nearly double the environmental contribution (5.15%) from enclosures. Key findings reveal (1) genotype-specific microbial signatures, notably G9’s enrichment of nutritional symbiont *Buchnera* and SCFA-producers, and G2’s association with *Bifidobacterium* spp. essential for exudativory; (2) a paradoxical reduction in microbial evenness under high polymorphism, suggesting trade-offs in immune-mediated microbial filtering; (3) enclosure-driven trade-offs where enriched Group III elevated beneficial *Lactobacillus* but concurrently increased pathogens (*Proteus*, *Corynebacterium*). These results demonstrate MHC–microbiota co-adaptation as a critical adaptive mechanism, advocating for genotype-targeted interventions: prioritizing ex situ breeding of high-heterozygosity individuals (e.g., G9 carriers), implementing allele-matched diets (e.g., gum arabic for G2 genotypes), and optimizing enclosure designs to balance microbial diversity with pathogen control. This integrated genetic–microbial framework significantly advances conservation strategies for this endangered primate.

## Figures and Tables

**Table 1:** Statistics of group information outside phylogenetic tree.

Closely Related Species	Gene Symbol	Genebank Number
*Nycticebus coucang*	Nyco-ESB	XM053603395
*Otolemur garnettii*	Otga-DRB1*1	XM012804740
*Otolemur garnettii*	Otga-DRB1*4	XM003789065
*Macaca mulatta*	Mamu-DRB5*0309	EU934778
*Bos taurus*	Bota-DRB3	BT029914
*Macaca nemestrina*	Mane-DRB*W001:12:01	LN998245
*Macaca fascicularis*	Mafa-DRB*1004	KR632829
*Colobus angolensis*	Coan-DRB1*4	XR_001002085.1
*Cerocebus atys*	Ceat-DRB3	XM012070072
*Homo sapiens*	HLA-DRB4	MW60513

**Table 2:** Polymorphism statistics of the Bengal slow loris *DRB1e2.*

	Totality	*I*	*II*	*III*
N	46	23	15	8
A	9	8	6	9
A_E_	5.71	5.48	3.98	7.41
A_R_	9	8	6	9
SNPs	91	39	10	42
PIS	84	42	25	22
PIC	0.82	0.82	0.75	0.87
H_E_	0.29	0.29	0.3	0.31
Tajima’s D	−0.21			
Pi	0.14	0.17	0.06	0.22
Fst	−0.02			
Over mean distance	0.15 ± 0.03	0.10 ± 0.05	0.07 ± 0.01	0.31 ± 0.05

Abbreviations: A, observed number of alleles; A_E_, effective number of alleles; A_R_, allelic richness; SNPs, single-nucleotide polymorphisms; PIS, polymorphic information site; PIC, polymorphic information content; H_E_, expected heterozygosity; Pi, nucleotide polymorphism; Fst, Fixation Index.

## Data Availability

The raw sequence data reported in this paper have been deposited in the Genome Sequence Archive (Genomics, Proteomics & Bioinformatics 2021) in National Genomics Data Center (Nucleic Acids Res 2022), China National Center for Bioinformation/Beijing Institute of Genomics, Chinese Academy of Sciences (GSA: CRA021047), and are publicly accessible at https://ngdc.cncb.ac.cn/gsa accessed on 5 December 2024.

## Supplementary Materials

The following supporting information can be downloaded at: https://www.mdpi.com/article/10.3390/biology14081094/s1, Figure S1: Comparison of ACE (A), Chao1 (B), Shannon (C), and PD_whole_tree (D) indices across different polymorphisms; Figure S2: Shannon and PD_whole_tree comparison under genotypes; Figure S3: PCoA (A,B) and NMDS (C,D) analysis of β diversity (weighted unifrac and unweighted unifrac distances) under different genotypes of *DRB1e2*; Figure S4: PCoA (A,B) and NMDS (C,D) analysis of β diversity (weighted unifrac and unweighted unifrac distances) under different polymorphisms of *DRB1e2*; Figure S5: Comparison of α diversity indices under enclosures; Figure S6: PCoA (A,B) and NMDS (C,D) analysis of β diversity (weighted unifrac and unweighted unifrac distances) under different enclosures; Figure S7: binary_jaccard.permanova.DistMatrixBoxplot under different enclosures; Table S1: *DRB1* allele frequencies and genotype frequencies in Bengal slow lorises under different enclosures; Table S2: The amino acid sequence of *DRB1e2* in Bengal slow lorises; Table S3: Genotype statistics of the Bengal slow loris *DRB1e2.*

## References

[1] Wang Y., Zhan H., Saif A., Zhang X., Su H. (2024). Analysis of winter survival strategies of sympatric black-necked cranes, and common cranes from the perspective of diet and gut microbiota. Ecol. Indic..

[2] Scholier T., Lavrinienko A., Kallio E.R., Watts P.C., Mappes T. (2024). Effects of past and present habitat on the gut microbiota of a wild rodent. Proc. R. Soc. B Biol. Sci..

[3] Larzul C., Estellé J., Borey M., Blanc F., Lemonnier G., Billon Y., Thiam M.G., Quinquis B., Galleron N., Jardet D. (2024). Driving gut microbiota enterotypes through host genetics. Microbiome.

[4] Bawden E.G., Wagner T., Schröder J., Effern M., Hinze D., Newland L., Attrill G.H., Lee A.R., Engel S., Freestone D. (2024). CD4^+^ T cell immunity against cutaneous melanoma encompasses multifaceted MHC II–dependent responses. Sci. Immunol..

[5] König R., Huang L.-Y., Germain R.N. (1992). MHC class II interaction with CD4 mediated by a region analogous to the MHC class I binding site for CD8. Nature.

[6] Kubinak J.L., Stephens W.Z., Soto R., Petersen C., Chiaro T., Gogokhia L., Bell R., Ajami N.J., Petrosino J.F., Morrison L. (2015). MHC variation sculpts individualized microbial communities that control susceptibility to enteric infection. Nat. Commun..

[7] Roland M.M., Mohammed A.D., Kubinak J.L. (2020). How MHCII signaling promotes benign host-microbiota interactions. PLoS Pathog..

[8] Fleischer R., Schmid D.W., Wasimuddin, Brändel S.D., Rasche A., Corman V.M., Drosten C., Tschapka M., Sommer S. (2022). Interaction between MHC diversity and constitution, gut microbiota and Astrovirus infections in a neotropical bat. Mol. Ecol..

[9] Montero B.K., Wasimuddin, Schwensow N., Gillingham M.A.F., Ratovonamana Y.R., Rakotondranary S.J., Corman V., Drosten C., Ganzhorn J.U., Sommer S. (2021). Evidence of MHC class I and II influencing viral and helminth infection via the microbiome in a non-human primate. PLoS Pathog..

[10] Fogel A.T. (2015). The Gut Microbiome of Wild Lemurs: A Comparison of Sympatric *Lemur catta* and *Propithecus verreauxi*. Folia Primatol..

[11] de Winter I.I., Qurkhuli T., de Groot N., de Vos-Rouweler A.J.M., van Hooft P., Heitkönig I.M.A., Prins H.H.T., Bontrop R.E., Doxiadis G.G.M. (2019). Determining Mhc-DRB profiles in wild populations of three congeneric true lemur species by noninvasive methods. Immunogenetics.

[12] Petersen R.M., Bergey C.M., Roos C., Higham J.P. (2022). Relationship between genome-wide and MHC class I and II genetic diversity and complementarity in a nonhuman primate. Ecol. Evol..

[13] Uren Webster T.M., Consuegra S., Hitchings M., Garcia de Leaniz C. (2018). Interpopulation Variation in the Atlantic Salmon Microbiome Reflects Environmental and Genetic Diversity. Appl. Environ. Microbiol..

[14] Couch C.E., Epps C.W. (2022). Host, Microbiome, and Complex Space: Applying Population and Landscape Genetic Approaches to Gut Microbiome Research in Wild Populations. J. Hered..

[15] Qingyong N., Xin H., Yu W., Xiangyun M., Nekaris K.A.I., Burrows A.M. (2020). Distribution and Conservation Status of Slow Lorises in Indo-China. Evolution, Ecology and Conservation of Lorises and Pottos.

[16] Thạch H.M., Le M.D., Vũ N.B., Panariello A., Sethi G., Sterling E.J., Blair M.E. (2018). Slow Loris Trade in Vietnam: Exploring Diverse Knowledge and Values. Folia Primatol..

[17] Qingyong N., Chen Z., Diyan L., Huailiang X., Yongfang Y., Mingwang Z., Xiaolan F., Bo Z., Deying Y., Meng X. (2021). Effects of Dietary Alteration on the Gut Microbiome and Metabolome of the Rescued Bengal Slow Loris. Front. Microbiol..

[18] McKenzie V.J., Song S.J., Delsuc F., Prest T.L., Oliverio A.M., Korpita T.M., Alexiev A., Amato K.R., Metcalf J.L., Kowalewski M. (2017). The Effects of Captivity on the Mammalian Gut Microbiome. Integr. Comp. Biol..

[19] Doxiadis G.G.M., Otting N., Groot N.G.d., Groot N.d., Rouweler A.J.M., Noort R., Verschoor E.J., Bontjer I., Bontrop R.E. (2003). Evolutionary stability of MHC class II haplotypes in diverse rhesus macaque populations. Immunogenetics.

[20] Song X., Zhang P., Huang K., Chen D., Guo S., Qi X., He G., Pan R., Li B. (2016). The influence of positive selection and trans-species evolution on DPB diversity in the golden snub-nosed monkeys (*Rhinopithecus roxellana*). Primates.

[21] Pierini F., Nutsua M., Böhme L., Özer O., Bonczarowska J., Susat J., Franke A., Nebel A., Krause-Kyora B., Lenz T.L. (2020). Targeted analysis of polymorphic loci from low-coverage shotgun sequence data allows accurate genotyping of HLA genes in historical human populations. Sci. Rep..

[22] Kennedy L.J., Ryvar R., Gaskell R.M., Addie D.D., Willoughby K., Carter S.D., Thomson W., Ollier W.E.R., Radford A.D. (2002). Sequence analysis of MHC DRB alleles in domestic cats from the United Kingdom. Immunogenetics.

[23] Rozas J., Ferrer-Mata A., Sánchez-DelBarrio J.C., Guirao-Rico S., Librado P., Ramos-Onsins S.E., Sánchez-Gracia A. (2017). DnaSP 6: DNA Sequence Polymorphism Analysis of Large Data Sets. Mol. Biol. Evol..

[24] Pers D., Hansen A.K. (2021). The boom and bust of the aphid’s essential amino acid metabolism across nymphal development. G3-Genes Genomes Genet..

[25] Cabana F., Clayton J.B., Nekaris K.A.I., Wirdateti W., Knights D., Seedorf H. (2019). Nutrient-based diet modifications impact on the gut microbiome of the Javan slow loris (*Nycticebus javanicus*). Sci. Rep..

[26] Khan M.A.W., Stephens W.Z., Mohammed A.D., Round J.L., Kubinak J.L. (2019). Does MHC heterozygosity influence microbiota form and function?. PLoS ONE.

[27] Clayton J.B., Vangay P., Huang H., Ward T., Hillmann B.M., Al-Ghalith G.A., Travis D.A., Long H.T., Tuan B.V., Minh V.V. (2016). Captivity humanizes the primate microbiome. Proc. Natl. Acad. Sci. USA.

[28] Reid G., Burton J. (2002). Use of Lactobacillus to prevent infection by pathogenic bacteria. Microbes Infect..

[29] Hoefer A., Seth-Smith H., Palma F., Schindler S., Freschi L., Dangel A., Berger A., D’Aeth J., Indra A., Fry N.K. (2023). Phenotypic and genomic analysis of a large-scale Corynebacterium diphtheriae outbreak among migrant populations in Europe. medRxiv.

