# Supplementary material for: The Role of MHC-II Diversity over Enclosure Design in Gut Microbiota Structuring of Captive Bengal Slow Lorises
Source: Biology (Basel). 2025 Aug 21;14(8):1094. doi: 10.3390/biology14081094 (PMC12383371; doi:10.3390/biology14081094)
Supplement: Supplementary file 1 [file biology-14-01094-s001.zip › biology-3789250-supplementary.pdf]

Supplementary Table S1 *DRB1* allele frequencies and genotype frequencies in Bengal slow lorises under different enclosures.

	**Allel gene**	***DRB1*01***	***DRB1*02***	***DRB1*03***	***DRB1*04***	***DRB1*05***	***DRB1*06***	***DRB1*07***	***DRB1*08***	***DRB1*09***		
**Totality**		**0.07**	**0.21**	**0.10**	**0.20**	**0.22**	**0.07**	**0.06**	**0.01**	**0.05**		
I		0.06	0.23	0.15	0.19	0.25	0.06	0.04	0	0.04		
II		0.08	0.29	0.03	0.26	0.31	0	0.03	0	0		
III		0.05	0.10	0.24	0.05	0.14	0.14	0.14	0.05	0.10		
	**Genetype**	**G1**	**G2**	**G3**	**G4**	**G5**	**G6**	**G7**	**G8**	**G9**	**G10**	**G11**
**Totality**		0.02	0.30	0.43	0.07	0.02	0.02	0.02	0.02	0.04	0.02	0.02
I		0	0.39	0.43	0.04	0.04	0	0	0	0.09	0	0
II		0.07	0.07	0.67	0.13	0	0	0.07	0	0	0	0
III		0	0.63	0	0	0	0.13	0	0.13	0	0.13	0

Note:In the table, "Totality" represents the gene frequency data of the total sample, and "I", "II", and "III" represent the gene frequency data of three different enclosures respectively; the left "Allele gene" column shows 9 DRB1 alleles (*DRB1*01* to *DRB1*09*), the "Genotype" column shows 11 genotypes (G1 to G11), and the values in the table are the frequencies of the corresponding genes or genotypes in different populations.

Supplementary Table S2 The amino acid sequence of *DRB1e2* in Bengal slow lorises.

Allel gene	Amino acid sequence					
***DRB1**01**	ERVRHLVR-D	LSPGGEGALR	QRRGGVPAGD	GAGAAGRRVL	EQPEGLPGAE	AGRGGHGVQTQ
***DRB1**02**	· · · · Y · · · -L	· Q · · ·V · · · ·	· · · · · · · · · ·	· · · · · · · · · ·	· · · · · ·H· · ·	· · · · · · · · · ·
***DRB1**03**	· · · · Y · · · -L	· Q · · ·VP · · ·	· · · · · · · · · ·	· · · · · · · · · ·	· · · · · H· · ·Q	.D.........
***DRB1**04**	· · · · Y · · · -L	· Q · · ·V · · · ·	· · · · · · · · · ·	· · · · · · · · · ·	· · · · · · · · ·D	· · · · · · · · · ·
***DRB1**05**	· · · · Y · · ·- ·	· Q · · ·VR · · ·	· · · · · · · · · ·	· · · · · · · · · ·	· · · · · · · · ·D	· · · · · · · · · ·
***DRB1**06**	· · · · Y · · ·P ·	· Q · · ·VR · · ·	· · · · · · · · · ·	· · · · · · · · · ·	· · · · · · · · ·D	· · · · · · · · · ·
***DRB1**07**	· · · · L· · · - ·	· · · · · VR · · ·	· · · · · · · · · ·	· · · · · · · · · ·	· · · · · · · · ·D	·D· · · · · · · ·
***DRB1**08**	· · · · F· · · -L	· Q · · ·VR · · ·	· · · · · · · · · ·	· · · · · ·D· ·L	· · · · · · · · ·H	· · · · · · ·H· ·
***DRB1**09**	· · · · F· · · -L	· · · · · VP · · ·	· · · · · · · · · ·	· · · · · · · · · ·	· · · · · · · · ·T	·D· · · · · · · ·

Note: The table takes the amino acid sequence of *DRB1*01* as a reference. Amino acids identical to those in *DRB1*01* in other alleles (*DRB1*02* to *DRB1*09*) are indicated by "·", and different amino acids show specific amino acid symbols or sequence differences, demonstrating the variation in amino acid sequences among different alleles.

Supplementary Table S3 Genotype statistics of the Bengal slow loris *DRB1e2*

**Genotype**	**Allel gene**	***N***
**G1**	*Nybe-DRB1*01*、*02*	1
**G2**	*Nybe-DRB1*03*	14
**G3**	*Nybe-DRB1*02*、*04*、*05*	20
**G4**	*Nybe-DRB1*01*、*02*、*05*	3
**G5**	*Nybe-DRB1*04*、*05*、*06*	1
**G6**	*Nybe-DRB1*02*、*04*、*05*、*06*、*07*	1
**G7**	*Nybe-DRB1*01*、*04*、*05*、*07*	1
**G8**	*Nybe-DRB1*08*	1
**G9**	*Nybe-DRB1*01*、*02*、*05*、*06*、*07*、*09*	2
**G10**	*Nybe-DRB1*05*、*06*、*07*、*09*	1
**G11**	*Nybe-DRB1*01*、*02*、*05*、*06*、*07*、*09*	1

Note: The left "Genotype" column lists 11 genotypes (G1 to G11), the "Allele gene" column corresponds to the combination of *DRB1* alleles contained in each genotype, and the "N" column indicates the number of samples with that genotype, reflecting the distribution scale of different genotypes in the population

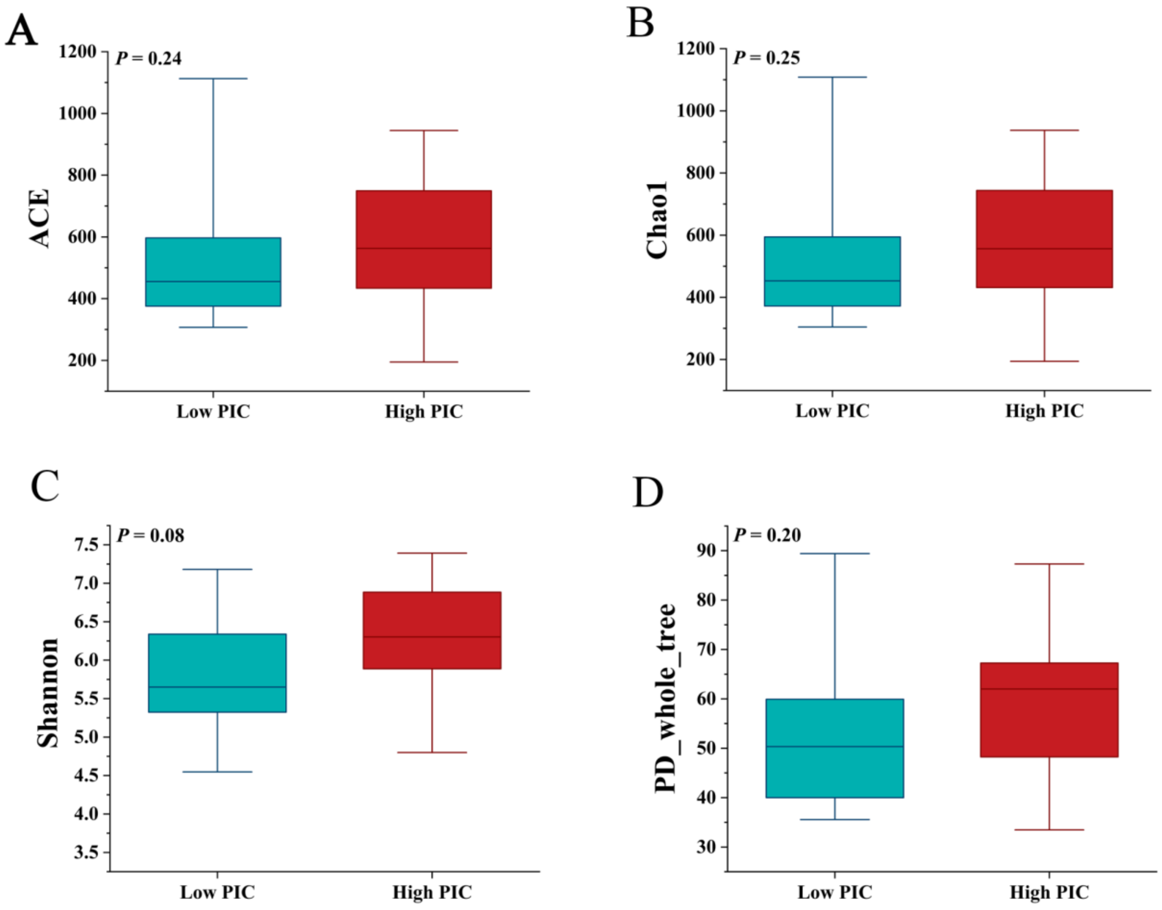

Supplementary Fig. S1 Comparison of ACE (A), Chao1 (B), Shannon (C), and PD_whole_tree (D) indices across different polymorphisms.

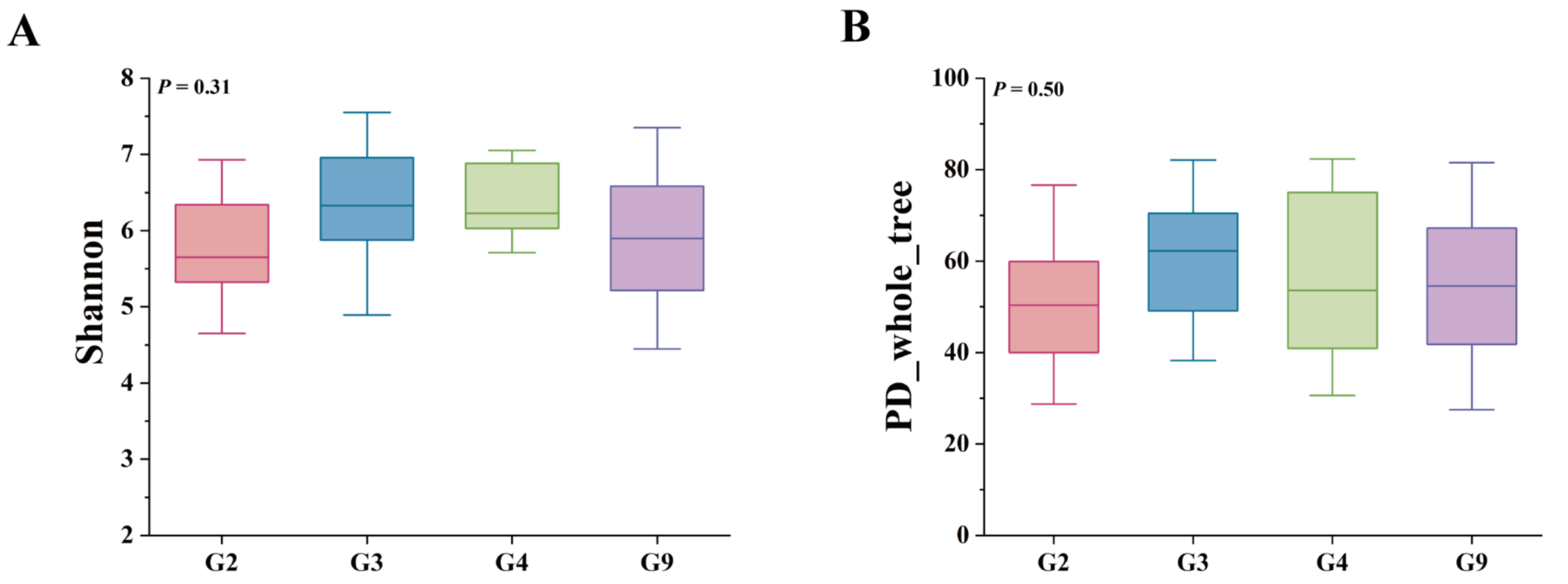

Supplementary Fig. S2 Shannon and PD_whole_tree comparison under genotypes. The abscissa in the chart represents different genotypes (G2, G3, G4, G9), and the ordinate represents the values of the Shannon index and PD_whole_tree index respectively. The P-values between different genotypes are marked above (e.g., P = 0.31 for the comparison of Shannon index between G2 and G3), which is used to show the differences in species diversity and phylogenetic diversity among different genotype populations and their statistical significance.

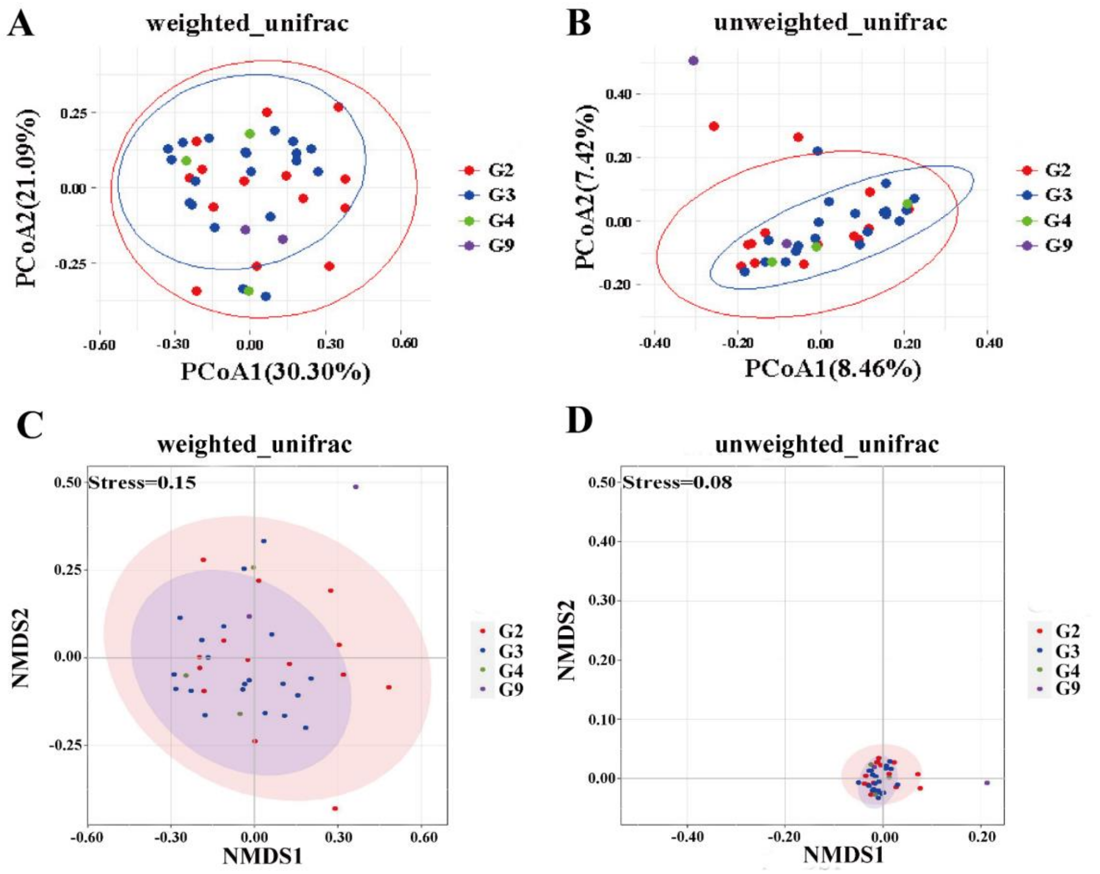

Supplementary Fig S3 PCoA (A, B) and NMDS (C, D) analysis of β diversity (weighted unifrac and unweighted unifrac distances) under different genotypes of *DRB1e2.* Among them, A and B are Principal Coordinate Analysis (PCoA) charts based on weighted UniFrac distance and unweighted UniFrac distance respectively, with the coordinate axes marked with the percentage of variation explained by the principal components (e.g., PCoA1 accounts for 30.30%); C and D are Non-metric Multidimensional Scaling (NMDS) charts marked with stress values (e.g., 0.15). Different points in the chart represent different samples, and the distribution distance of samples in the chart reflects the differences in microbial community composition among different genotype populations.

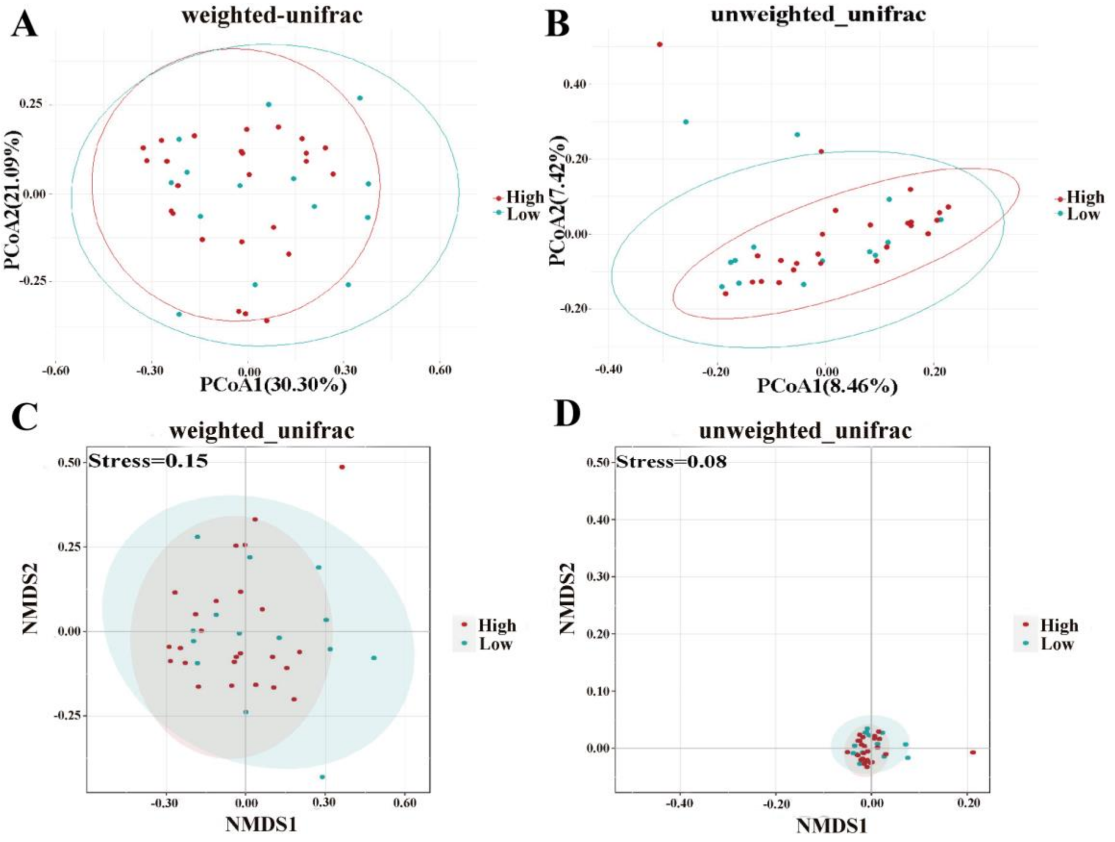

Supplementary Fig S4 PCoA (A, B) and NMDS (C, D) analysis of β diversity (weighted unifrac and unweighted unifrac distances) under different polymorphisms of *DRB1e2.* A and B are the results of PCoA analysis corresponding to weighted and unweighted UniFrac distances respectively, showing the distribution of microbial community structures of samples under different polymorphism levels in the principal component space; C and D are the results of NMDS analysis, reflecting the fitting degree of the analysis through stress values. The distribution distance of sample points reflects the differences in microbial community composition among different polymorphism populations.

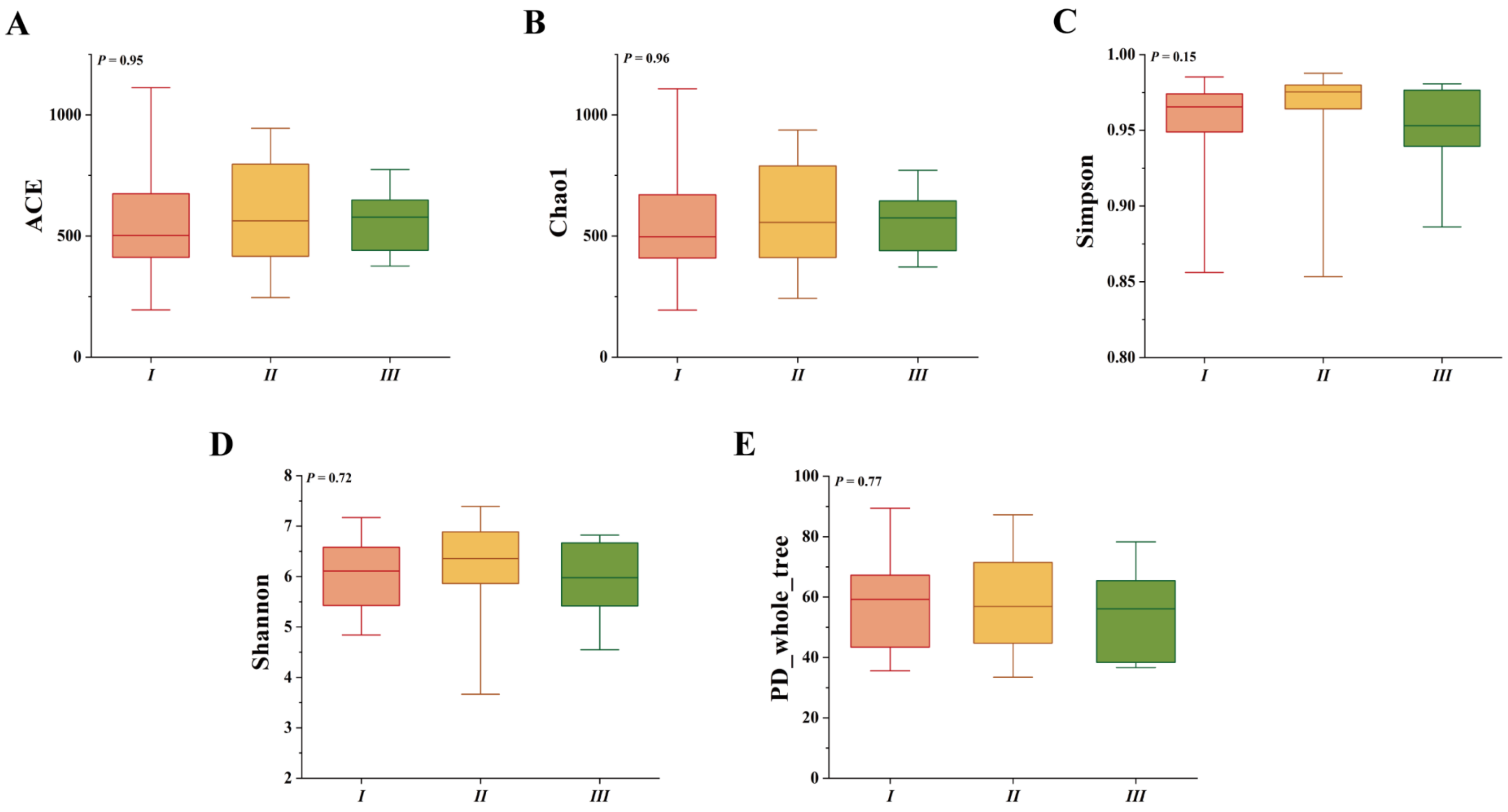

Supplementary Fig S5 Comparison of α diversity indices under enclosures. A, B, and C in the chart show Chao1 index and ACE index respectively, which are used

to reflect species richness; D and E show the Shannon index, which is used to reflect species diversity. The P-values between different enclosures are marked in the chart (e.g., P=0.15 for the comparison of Chao1 index between I and II), reflecting the impact of different enclosures on the α diversity of microbial communities and their statistical significance.

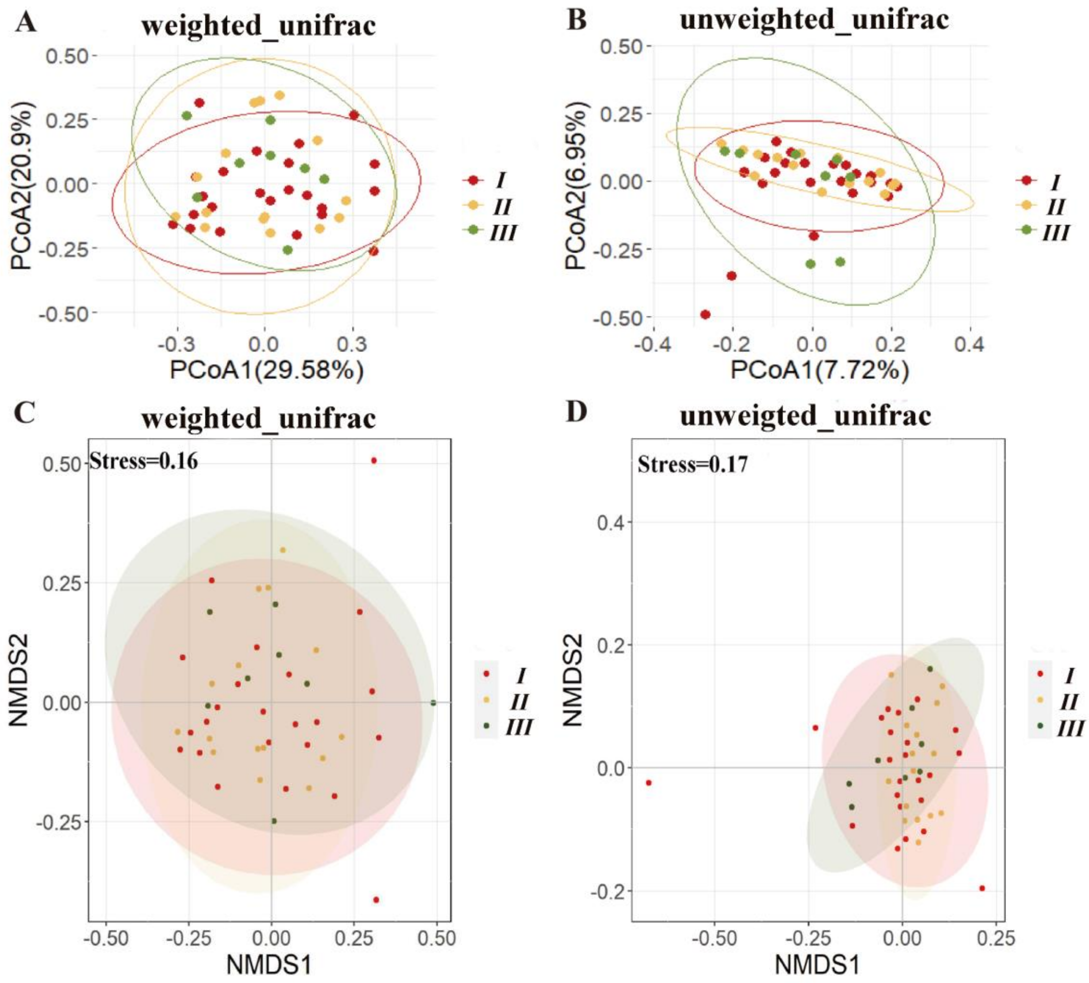

Supplementary Fig S6 PCoA (A, B) and NMDS (C, D) analysis of β diversity (weighted unifrac and unweighted unifrac distances) under different enclosures. A and B are PCoA analyses based on weighted and unweighted UniFrac distances respectively, showing the sample distribution through the percentage of variation explained by the principal components (e.g., PCoA1); C and D are NMDS analyses marked with stress values. The degree of aggregation or dispersion of sample points in the chart reflects the similarity or difference in microbial community composition under different enclosures.

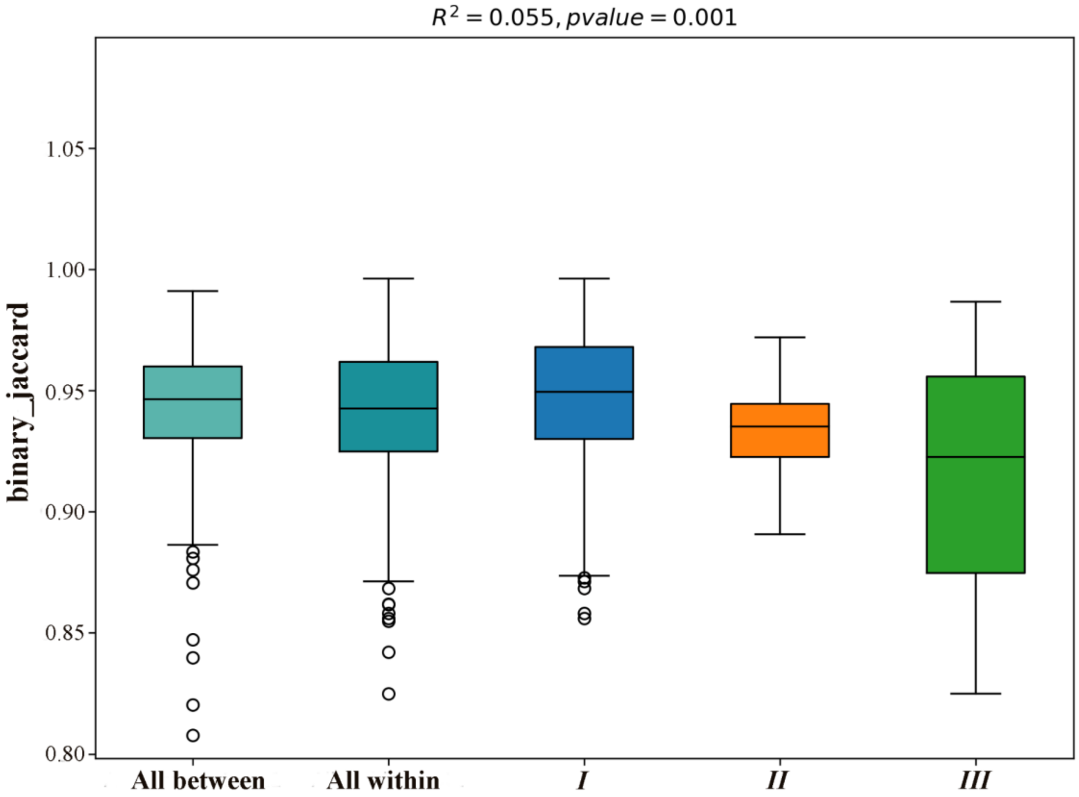

Supplementary Fig S7 binary_jaccard.permanova.DistMatrixBoxplot under different enclosures. The abscissa in the chart is divided into "All between" (samples between different enclosures) and "All within" (samples within the same enclosure), and the ordinate is the distance value. The boxplot shows the difference in distance distribution between the two types of samples. The coefficient of determination (R^2=0.055) and P-value (pvalue=0.001) are marked in the chart, which are used to illustrate the explanatory power of enclosures on the variation of microbial community composition and the statistical significance of this difference.
